# Comparison of body mass index and fat mass index to classify body composition in adolescents—The EVA4YOU study

**DOI:** 10.1007/s00431-024-05474-x

**Published:** 2024-02-22

**Authors:** Alex Messner, Johannes Nairz, Sophia Kiechl, Bernhard Winder, Raimund Pechlaner, Ralf Geiger, Michael Knoflach, Ursula Kiechl-Kohlendorfer, for the EVA4YOU Tyrol Study Group, Mandy Asare, Manuela Bock-Bartl, Alexander E. Egger, Ralf Geiger, Silvia Gelmi, Andrea Griesmacher, Christoph Hochmayr, Jonas Huber, Sophia J. Kiechl, Stefan Kiechl, Ursula Kiechl-Kohlendorfer, Michael Knoflach, Alex Laner, Denise Lazzarotto, Alex Messner, Johannes Nairz, Hannah Oberhammer, Raimund Pechlaner, Bernhard Winder

**Affiliations:** 1 VASCage, Centre on Clinical Stroke Research, Innsbruck, Austria; 2 Department of Pediatrics II, Medical University of Innsbruck, Anichstraße 35, 6020 Innsbruck, Austria; 3 Department of Pediatrics III, Medical University of Innsbruck, Innsbruck, Austria; 4 Department of Neurology, Hochzirl Hospital, Zirl, Austria; 5 Department of Vascular Surgery, Feldkirch Hospital, Feldkirch, Austria; 6 Department of Neurology, Medical University of Innsbruck, Anichstraße 35, 6020 Innsbruck, Austria; 7 Central Institute of Clinical Chemistry and Laboratory Medicine (ZIMCL), Medical University of Innsbruck, Innsbruck, Austria; 8 Department of Vascular Surgery, Landeskrankenhaus Feldkirch, Feldkirch, Austria

**Keywords:** Adolescents, Overweight, Fat mass index, Fat-free mass index, Body mass index, Bioelectrical impedance analysis

## Abstract

The objectives of this study were to develop age- and sex-specific reference percentiles for fat mass index (FMI) and fat-free mass index (FFMI) in adolescents aged 14 to 19 years and to determine differences in overweight/obesity classification by FMI and body mass index (BMI). The EVA4YOU study is a single-center cross-sectional study conducted in western Austria. Cardiovascular risks including anthropometric measurements and bioelectrical impedance analysis were assessed in adolescents (mean age 17 years). FMI and FFMI were calculated as the ratio of fat mass (FM) and fat-free mass (FFM) to the square of height and compared to study population–specific BMI percentiles. One thousand four hundred twenty-two adolescents were included in the analysis. Girls had a significantly higher mean FM and FMI and a significantly lower mean FFM, FFMI (*p* < 0.001, each), and mean BMI (*p* = 0.020) than boys. Body composition classification by FMI and BMI percentiles shows a concordance for the < 75th and > 97th percentile, but a significant difference in percentile rank classifications between these two cut-off values (all *p* < 0.05). Based on FMI, 15.5% (221/1422) of the whole population and 29.4% (92/313) of those between the 75th and 97th percentiles are classified one category higher or lower than those assigned by BMI.

*Conclusion*: Classification of normal or pathologic body composition based on BMI and FMI shows good accordance in the clearly normal or pathologic range. In an intermediate range, FMI reclassifies categories based on BMI in more than a quarter of adolescents. Cut-off values to differentiate normal from pathologic FMI values on a biological basis are needed.

*Trial registration*: The study is registered at www.clinicaltrials.gov (Identifier: NCT04598685; Date of registration: October 22, 2020).

**Table:** 

**What is Known:** *• Chronic non-communicable diseases (NCDs) are the leading cause of morbidity and mortality globally, with major risk factors including unhealthy diets, harmful behaviors, and obesity. Obesity in children and adolescents is a key risk factor for later NCDs, which is commonly measured by Body Mass Index (BMI).* *• BMI can be misleading as it doesn't distinguish between fat mass (FM) and fat-free mass (FFM), leading to potential misclassification of obesity in children. Previous studies have already suggested the use of the Fat Mass Index (FMI) and Fat-Free Mass Index (FFMI) as a more accurate measures of body composition.*	
**What is New:** *• This study adds the first age- and sex-specific reference values for FMI and FFMI in Austrian adolescents using bioelectrical impedance analysis (BIA) as a safe and secure measurement method of a large representative cohort.* *• We found percentile misclassification between BMI and FMI when categorizing for obesity, especially in intermediate categories of body composition. Furthermore, when comparing the new reference values for FMI and FFMI to existing ones from the US, UK, and Germany we could show a good alignment within the European cohorts and major differences with American values, indicating and confirming the difference of FMI and FFMI for different populations of different ethnical background, living on different continents.*

**What is Known:**

*• Chronic non-communicable diseases (NCDs) are the leading cause of morbidity and mortality globally, with major risk factors including unhealthy diets, harmful behaviors, and obesity. Obesity in children and adolescents is a key risk factor for later NCDs, which is commonly measured by Body Mass Index (BMI).*

*• BMI can be misleading as it doesn't distinguish between fat mass (FM) and fat-free mass (FFM), leading to potential misclassification of obesity in children. Previous studies have already suggested the use of the Fat Mass Index (FMI) and Fat-Free Mass Index (FFMI) as a more accurate measures of body composition.*

**What is New:**

*• This study adds the first age- and sex-specific reference values for FMI and FFMI in Austrian adolescents using bioelectrical impedance analysis (BIA) as a safe and secure measurement method of a large representative cohort.*

*• We found percentile misclassification between BMI and FMI when categorizing for obesity, especially in intermediate categories of body composition. Furthermore, when comparing the new reference values for FMI and FFMI to existing ones from the US, UK, and Germany we could show a good alignment within the European cohorts and major differences with American values, indicating and confirming the difference of FMI and FFMI for different populations of different ethnical background, living on different continents.*

## Introduction

Chronic non-communicable diseases (NCDs) are nowadays the main cause of morbidity and mortality worldwide. The major risk factors for the development of NCDs are unhealthy diets; harmful behavioral patterns such as smoking, heavy alcohol consumption, and lack of exercise; and obesity [1–3]. When considering children and adolescents, obesity is one of the most important risk factors for later NCDs [4–6]. BMI is widely used to identify excess adiposity, both in adults and in adolescents [7]. However, it is not increased body weight that is crucial for the development of such diseases, but excessive body fat [8–10]. Body mass index (BMI) as a sole measure of obesity can lead to misclassification, because actual body fat is not measured and fat mass (FM) cannot be distinguished from lean mass, especially in childhood [11]. This can result in a misclassification of children with a high amount of lean mass as overweight or obese and of children with a high amount of body fat and little lean mass as normal weight [12–15]. Other pediatric studies in various populations came to the same conclusion when comparing the percentage body fat with BMI as a surrogate for adiposity [16]. Therefore, a measure of the fat mass itself is useful in an exact classification. In order to avoid ambiguities in absolute values or percentages, it is recommended to use height-specific indexes of FM and fat-free mass (FFM): the fat mass index (FMI) and the fat-free mass index (FFMI) [17].

Recent studies have published FMI and FFMI percentile curves for children and adolescents [18–21]. Reference values by Wells et al. were generated using the 4-compartment model, Weber et al. used dual-energy X-ray absorptiometry in a representative American cohort (National Health and Nutrition Examination Survey, NHANES) [18, 19]. Another simple, non-invasive, non-radiative, and cost-effective method to assess body composition in adults and children is bioelectrical impedance analysis (BIA), which has already been used to generate reference percentiles for adult and also pediatric populations [20–24]. So far, there exist FMI and FFMI reference values for American (NHANES), British (volunteers from south-east England), and German (northern city of Kiel) children and adolescents with the possibility to convert measured values into exact *z*-scores [18, 19, 21]. No reference percentiles exist for adolescents living in central European Alpine regions.

The purpose of this study was to develop the first age- and sex-specific reference values for FMI and FFMI in Austria using non-invasive BIA in adolescents aged 14 to 19 years from the Early Vascular Ageing in the YOUth (EVA4YOU) study and to compare differences in percentile classification when applying FMI or BMI.

## Methods

### Participants

This study is part of the EVA4YOU study, which is a single-center cross-sectional study conducted at the Department of Neurology and the Department of Pediatrics at the Medical University of Innsbruck. The aim of the EVA4YOU study is to assess cardiovascular risk profiles in adolescents aged 14–19 years in the central European region of Tyrol, Austria.

The study was conducted directly at schools and workplaces in Tyrol, Austria, from January 2021 to March 2023. All schools as well as large companies were invited to participate and contacted in person, whereby attention was paid to achieve a representative distribution over whole Tyrol. After an informational presentation about early vascular aging and the scope and procedures of the study to interested students and vocational trainees in schools and workplaces, written informed consent was obtained by adolescents and their legal guardian in adolescents aged < 18 years. Clinical examination took place at school or at work, followed by discussion of the results with each subject individually by a medical doctor a few weeks later.

The study-specific examinations were adapted from the EVA Tyrol study (NCT03929692) [25] and included a broad assessment of vascular risk factors and behaviors and a detailed vascular phenotyping as well as standard lab values. For the present analysis, we included information on age, sex, self-reported ethnicity, and body composition as well as the socioeconomic status.

Socioeconomic status was assessed by the Family Affluence Scale score [26], an index of family wealth, ranging from 0 to 9 points. A score of 0–2 points was classified as low, 3–5 points as medium, and 6–9 points as high socioeconomic status.

### Evaluation of body composition

Anthropometric measurements included weight (kg) and height (m). Subjects were weighted barefoot with light clothing to the nearest 0.1 kg with a portable electronic digital scale (Soehnle style sense compact 200, Backnang, Germany). Body height was measured using a mounted meter scale in classrooms or workplaces to the nearest 0.001 m. BIA resistance (Ω), reactance (Ω), and phase angle (calculated as the (reactance/resistance) × (180°/π)) (°) at 50 kHz were measured using Akern BIA 101 BIVA (Akern s.r.l., Florence, Italy).

Participants did not exercise on the day of the study, were barefoot, and did not wear metallic conductive wearables on their bodies. Measurement was taken after a period of rest in supine position on a non-conductive surface with hands extended away from the trunk and the feet apart. Disposable adhesive AgCl gel electrodes were placed on the dorsal sites of the ankle, foot, wrist, and hand on the right side of the body and connected to the device using guide cables. BIA measurements were performed by specially trained (at least 40 supervised measurements) medical staff according to the manufacturer’s instructions.

BMI was calculated based on body height and weight with the formula BMI = body weight (kg)/square of body height (m^2^). Based on the measured height and FM obtained by BIA, the FMI was calculated using the formula FMI = FM (kg)/square of body height (m^2^). FFMI was calculated using measured height and FFM obtained by BIA, using the formula FFMI = FFM (kg)/square of body height (m^2^).

### Generation of reference curves

We calculated age- and sex-specific reference curves for FMI, FFMI, and BMI for adolescents aged 14–19 years using the computer program RefCurv (version 0.4.2; Winkler 2020, https://refcurv.com). RefCurv is a special software package for generating pediatric reference curves, using R and the Generalized Additive Models for Location Scale and Shape (GAMLSS) add-on package as the underlying statistical engine. The GAMLSS and Lambda-Mu-Sigma (LMS) methods are commonly used approaches for creating reference percentiles, as they correct for the heteroscedasticity and skewness often present in growth data [27, 28]. In the LMS method, the degrees of freedom for splines L, M, and S have a great impact on the smoothness of the resulting percentile curves. The degrees of freedom of the hyperparameters L, M and S were determined using a grid search based on the Bayesian information criterion (BIC), selecting the degrees of freedom of the three parameters with the lowest BIC. Percentile curves were calculated and plotted using the RefCurv model fit function [29].

*z*-scores were calculated for each subject using the formula [(*X*/M)^L^ − 1]/LS where *X* is the measurement of interest.

As international [30] and national [31] reference values utilize different cut-offs for BMI classifying overweight and obesity and there are no firmly established cut-offs for FMI (≥ 75th was suggested by Weber et al. [18]), we decided to compare FMI and BMI using various categories with cut-offs between the 75th and 97th population-specific percentiles. Subjects were classified into the associated percentiles based on their BMI and FMI *z*-scores (< 75th percentile, 75th– < 85th percentile, 85th– < 90th percentile, 90th– < 95th percentile, 95th– < 97th percentile, ≥ 97th percentile).

### Statistical methods

Data analysis was performed using SPSS Statistics 29 (IBM, New York, USA). Obtained data were checked for integrity, methodological errors, and outliers. Normal distribution was assessed graphically using P-P plots and histograms. Potential independent variables included age, weight, height, FM, FFM, BMI, FMI, FFMI, and sex. For normal distribution, comparison of two groups was performed using a *t*-test. When homogeneity of variances was not given, Welch’s *F* was interpreted instead. When data were not normal distributed, Mann-Whitney *U* test was used to compare two groups. When comparing more than two groups, ANCOVA was performed, adjusted for sex and age. When groups were not normally distributed, bias-corrected and accelerated confidence intervals and *p*-values were evaluated. A two-tailed *p* < 0.05 was considered statistically significant.

## Results

### Study population

A total of 3940 adolescents participated at the study presentations at schools and companies of which a total of 1701 adolescents volunteered to take part in the study. One hundred eighty-four did not meet the inclusion criteria; informed consent was missing by either them or their legal guardian (if the subject was < 18 years old at the time of examination) or did not show up on the day of the examination (mainly due to sickness). In 95 cases, BIA measurements were missing or inadmissible, leaving 1422 adolescents for the present analysis.

We were able to collect socioeconomic status information from 99.4% (*n* = 1413) of respondents. In our study population, 0.4% (*n* = 6) reported poor, 19.8% (*n* = 280) medium, and 79.8% (*n* = 1127) high socioeconomic status. Information about ethnic origin was obtained in 99.6% (*n* = 1417) of participants. 98.7% (*n* = 1399) of them reported being Caucasian. 1.3% (*n* = 18) reported having a different ethnicity.

### Sex-specific differences and generation of FMI and FFMI reference values

In total, a number of 1422 adolescents were eligible for the generation of FMI and FFMI reference values. 63.4% (*n* = 902) were female participants and the mean age was 17.2 ± 1.3 years (girls: 17.2 ± 1.3 years, boys: 17.2 ± 1.3).

**Fig. 1:**
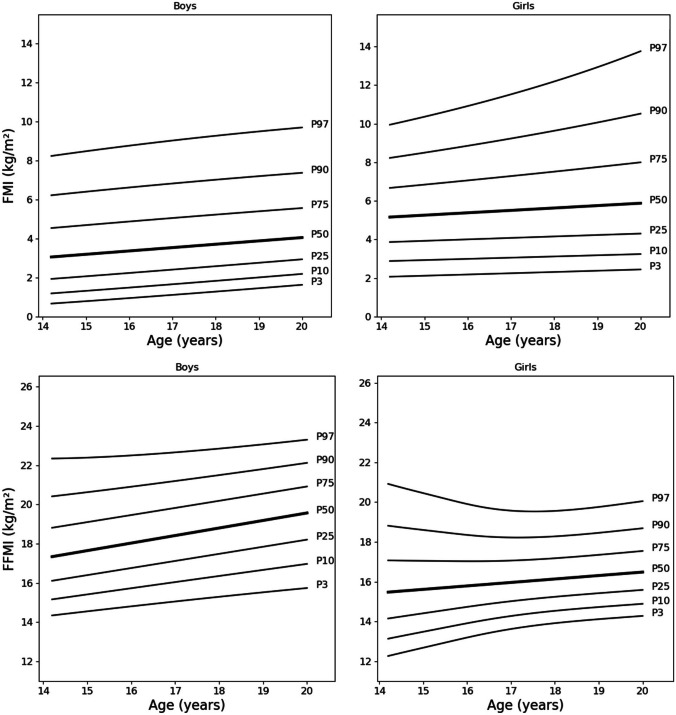
Reference curves for FMI and FFMI in boys and girls aged 14 to 19 years (3rd, 10th, 25th, 50th, 75th, 90th, and 97th percentile is shown)

BMI in boys was on average 0.47 kg/m^2^ higher than in girls (*p* = 0.002). Girls had a significantly higher mean FM of 3.65 kg and a higher mean FMI of 1.93 kg/m^2^, but a significantly lower mean FFM of 14.91 kg and a lower mean FFMI of 2.42 kg/m^2^ than boys (*p* < 0.001, each) (Table 1). Therefore, the reference percentiles for FMI and FFMI were calculated for boys and girls separately as in precedent studies [18, 19]. Smoothed reference percentiles for FMI and FFMI for boys and girls aged 14–19 years are shown in Table 2. In addition to the 50th percentile (M), each table also includes L and S values that can be used to calculate *z* scores for individuals. Growth curves indicating the 5th, 10th, 25th, 50th, 75th, 90th, and 97th percentiles for FMI and FFMI in boys and girls are shown in Fig. 1.

**Table 1:** Descriptive information on the EVA4YOU study population

	Boys	Girls	Mean difference	*p*-value
	(*n* = 520)	(*n* = 902)		
Age (years)	17.2 ± 1.3	17.2 ± 1.3	0.1 (− 0.2; 0.1)	0.289^a^
BMI (kg/m^2^)	22.6 ± 3.5	22.1 ± 3.7	0.5 (0.1; 0.9)	0.002^b^
FM (kg)	12.8 ± 7.0	16.5 ± 7.4	3.7 (2.9; 4.5)	< 0.001^b^
FMI (kg/m^2^)	4.0 ± 2.2	5.9 ± 2.6	1.9 (1.7; 2.2)	< 0.001^b^
FFM (kg)	59.7 ± 8.2	44.8 ± 5.3	14.9 (14.1; 15.7)	< 0.001^b^
FFMI (kg/m^2^)	18.6 ± 2.0	16.2 ± 1.7	2.4 (2.2; 2.6)	< 0.001^b^

Values are presented as mean ± SD and mean difference between sex (95% CI)

*BMI* body mass index, *FM* fat mass, *FMI* fat mass index, *FFM* fat-free mass, *FFMI* fat-free mass index, *CI* confidence interval

^a^Student *t* test

^b^Mann-Whitney *U* test

**Table 2:** Age- and sex-specific reference percentiles for FMI and FFMI in Tyrolean adolescents aged 14–19 years for boys and girls

		Boys									Girls								
	Age	L	S	3rd	10th	25th	50th (M)	75th	90th	97th	L	S	3rd	10th	25th	50th (M)	75th	90th	97th
FMI	14.0	0.386	0.619	0.6	1.2	1.9	3.0	4.5	6.1	8.0	0.428	0.401	2.1	2.9	3.9	5.1	6.6	8.2	9.9
	14.5	0.368	0.611	0.7	1.2	2.0	3.1	4.5	6.2	8.1	0.408	0.403	2.1	2.9	3.9	5.2	6.7	8.3	10.0
	15.0	0.336	0.596	0.8	1.3	2.1	3.2	4.6	6.3	8.2	0.373	0.408	2.1	2.9	3.9	5.2	6.8	8.5	10.3
	15.5	0.308	0.583	0.9	1.4	2.1	3.3	4.7	6.4	8.4	0.342	0.413	2.2	3.0	4.0	5.3	6.9	8.6	10.6
	16.0	0.276	0.569	0.9	1.5	2.2	3.4	4.8	6.5	8.6	0.307	0.418	2.2	3.0	4.0	5.4	7.0	8.8	10.9
	16.5	0.248	0.556	1.0	1.6	2.3	3.4	4.9	6.6	8.7	0.276	0.422	2.2	3.0	4.0	5.4	7.1	9.0	11.1
	17.0	0.216	0.543	1.1	1.7	2.4	3.5	5.0	6.7	8.9	0.241	0.427	2.3	3.1	4.1	5.5	7.3	9.2	11.5
	17.5	0.187	0.531	1.2	1.7	2.5	3.6	5.1	6.9	9.0	0.210	0.432	2.3	3.1	4.1	5.6	7.4	9.4	11.8
	18.0	0.156	0.518	1.3	1.8	2.6	3.7	5.2	7.0	9.2	0.175	0.437	2.3	3.1	4.2	5.6	7.5	9.6	12.1
	18.5	0.127	0.507	1.4	1.9	2.7	3.8	5.3	7.1	9.3	0.144	0.442	2.3	3.1	4.2	5.7	7.6	9.8	12.5
	19.0	0.095	0.495	1.5	2.0	2.8	3.9	5.4	7.2	9.4	0.109	0.447	2.4	3.2	4.2	5.7	7.7	10.0	12.9
	19.5	0.067	0.484	1.5	2.1	2.8	4.0	5.5	7.3	9.6	0.078	0.452	2.4	3.2	4.3	5.8	7.8	10.2	13.2
	20.0	0.035	0.472	1.6	2.2	2.9	4.0	5.6	7.4	9.7	0.043	0.457	2.4	3.2	4.3	5.9	8.0	10.5	13.7
FFMI	14.0	− 0.634	0.109	14.3	15.2	16.1	17.3	18.7	20.1	21.6	− 0.871	0.138	12.2	13.1	14.1	15.5	17.0	18.7	20.7
	14.5	− 0.557	0.108	14.4	15.3	16.2	17.5	18.8	20.2	21.7	− 0.922	0.132	12.4	13.3	14.2	15.5	17.0	18.6	20.6
	15.0	− 0.419	0.108	14.5	15.4	16.4	17.7	19.0	20.4	21.8	− 1.014	0.123	12.7	13.5	14.4	15.6	17.0	18.5	20.3
	15.5	− 0.297	0.108	14.6	15.6	16.6	17.8	19.2	20.5	22.0	− 1.096	0.114	12.9	13.7	14.6	15.7	17.0	18.4	20.1
	16.0	− 0.159	0.107	14.8	15.7	16.8	18.0	19.4	20.7	22.1	− 1.187	0.106	13.2	13.9	14.7	15.8	17.0	18.3	19.8
	16.5	− 0.037	0.107	14.9	15.9	16.9	18.2	19.6	20.9	22.3	− 1.269	0.099	13.4	14.1	14.9	15.9	17.0	18.2	19.6
	17.0	0.101	0.107	15.0	16.0	17.1	18.4	19.8	21.1	22.5	− 1.361	0.094	13.6	14.3	15.0	16.0	17.0	18.2	19.5
	17.5	0.224	0.106	15.2	16.2	17.3	18.6	20.0	21.3	22.6	− 1.442	0.090	13.8	14.4	15.1	16.0	17.1	18.2	19.5
	18.0	0.362	0.106	15.3	16.4	17.5	18.8	20.2	21.4	22.8	− 1.534	0.088	13.9	14.5	15.2	16.1	17.2	18.3	19.5
	18.5	0.484	0.105	15.4	16.5	17.6	19.0	20.3	21.6	22.9	− 1.616	0.087	14.0	14.6	15.3	16.2	17.3	18.3	19.6
	19.0	0.622	0.105	15.5	16.7	17.8	19.2	20.5	21.8	23.1	− 1.708	0.087	14.1	14.7	15.4	16.3	17.4	18.5	19.8
	19.5	0.744	0.105	15.6	16.8	18.0	19.3	20.7	22.0	23.3	− 1.789	0.087	14.2	14.8	15.5	16.4	17.4	18.6	19.9
	20.0	0.882	0.104	15.8	17.0	18.2	19.5	20.9	22.2	23.4	− 1.881	0.088	14.3	14.9	15.6	16.5	17.6	18.7	20.1

*FMI* fat mass index, *FFMI* fat-free mass index, *L* (lambda) optimal power to obtain normality, *M* (mu) median, *S* (sigma) coefficient of variation

### Comparison between our newly generated FMI and FFMI percentiles and already existing reference values

When comparing the percentile values derived from our study population to those from the US, England, and Germany, several differences became evident. The most notable disparities were observed in comparison to the values reported by Weber et al. for American adolescents. The FMI median (50th percentile) differed on average by 1.5 kg/m^2^ (range 1.2–1.9, depending on age group) in boys and 2.2 kg/m^2^ (range 1.7–2,7) in girls whereas the FFMI median (50th percentile) differed at a mean of − 1.3 kg/m^2^ (range – 1.0 to − 2.1) in boys and − 1.4 kg/m^2^ (range − 1.3 to − 1.5) in girls [18]. Very little difference in the comparison to the percentiles of the British [19] and German [21] cohorts was found (Table 3).

**Table 3:** Comparative analysis of FMI and FFMI values for different percentiles between American, British, and German reference studies and the EVA4YOU study

			Boys			Girls		
		Age (years)	50th P	75th P	90th P	50th P	75th P	90th P
Weber et al. [18]	FMI	14.0	1.9	2.3	3.6	1.7	2.4	3.7
		14.5	1.8	2.3	3.5	1.8	2.5	3.8
		15.0	1.7	2.2	3.5	1.9	2.5	3.8
		15.5	1.6	2.1	3.4	1.9	2.6	3.9
		16.0	1.5	2.0	3.3	2.0	2.7	4.0
		16.5	1.5	1.9	3.2	2.1	2.8	4.0
		17.0	1.4	1.9	3.2	2.2	2.8	4.1
		17.5	1.3	1.8	3.1	2.2	2.9	4.2
		18.0	1.3	1.8	3.0	2.4	3.0	4.3
		18.5	1.2	1.8	3.1	2.4	3.1	4.5
		19.0	1.2	1.8	3.1	2.6	3.3	4.6
		19.5	1.2	1.8	3.2	2.7	3.4	4.7
		20.0	1.3	1.9	3.3	2.7	3.5	4.7
	Mean difference		1.5	2.0	3.3	2.2	2.9	4.2
	FFMI	14.0	− 2.1	− 1.9	− 1.6	− 1.4	− 1.5	− 1.7
		14.5	− 1.8	− 1.5	− 1.1	− 1.3	− 1.4	− 1.4
		15.0	− 1.5	− 1.2	− 0.8	− 1.3	− 1.3	− 1.2
		15.5	− 1.2	− 1.0	− 0.5	− 1.4	− 1.2	− 1.0
		16.0	− 1.1	− 0.8	− 0.3	− 1.4	− 1.1	− 0.8
		16.5	− 1.0	− 0.7	− 0.2	− 1.4	− 1.1	− 0.7
		17.0	− 1.0	− 0.7	− 0.2	− 1.5	− 1.0	− 0.6
		17.5	− 1.0	− 0.7	− 0.1	− 1.4	− 1.1	− 0.5
		18.0	− 1.1	− 0.7	0.0	− 1.4	− 1.1	− 0.5
		18.5	− 1.1	− 0.7	− 0.1	− 1.5	− 1.1	− 0.5
		19.0	− 1.2	− 0.8	− 0.1	− 1.5	− 1.1	− 0.6
		19.5	− 1.2	− 0.9	− 0.2	− 1.5	− 1.0	− 0.6
		20.0	− 1.4	− 1.0	− 0.3	− 1.5	− 1.2	− 0.6
	Mean difference		− 1.3	− 1.0	− 0.4	− 1.4	− 1.2	− 0.8
Wells et al. [19]	FMI	14.0	0.0	− 0.1	0.7	0.2	0.5	1.5
		15.0	− 0.3	− 0.4	0.3	0.3	0.5	1.4
		16,0	− 0.6	− 0.6	0.0	0.3	0.5	1.2
		17.0	− 0.6	− 0.8	0.0	0.4	0.4	0.9
		18.0	− 0.6	− 0.6	0.3	0.5	0.3	0.6
		19.0	− 0.4	− 0.2	1.0	0.5	0.3	0.3
		20.0	0.0	0.3	1.8	0.5	0.1	− 0.2
	Mean difference		− 0.3	− 0.4	0.6	0.4	0.4	0.8
	FFMI	14.0	− 1.5	− 1.8	− 1.9	− 0.7	− 1.2	− 1.7
		15.0	− 1.1	− 1.2	− 1.3	− 0.5	− 0.8	− 1.1
		16.0	− 0.7	− 0.9	− 0.8	− 0.5	− 0.6	− 0.7
		17.0	− 0.5	− 0.7	− 0.7	− 0.6	− 0.6	− 0.6
		18.0	− 0.4	− 0.6	− 0.5	− 0.7	− 0.8	− 0.8
		19.0	− 0.5	− 0.5	− 0.6	− 0.9	− 1.1	− 1.1
		20.0	− 0.4	− 0.7	− 0.8	− 1.2	− 1.4	− 1.5
	Mean difference		− 0.8	− 0.9	− 0.9	− 0.7	− 0.9	− 1.1
Gätjens et al. [21]	FMI	14.0	0.5		0.8	− 0.2		0.0
		14.5	0.4		0.7	− 0.1		0.1
		15.0	0.2		0.6	0.1		0.1
		15.5	0.2		0.6	0.2		0.3
		16.0	0.1		0.7	0.2		0.4
		16.5	0.1		0.7	0.4		0.5
		17.0	0.0		0.8	0.4		0.7
	Mean difference		0.2		0.7	0.1		0.3
	FFMI	14.0	− 1.1		− 1.7	− 0.4		− 1.7
		14.5	− 0.9		− 1.4	− 0.3		− 1.4
		15.0	− 0.8		− 1.1	− 0.2		− 1.1
		15.5	− 0.6		− 0.9	− 0.1		− 0.8
		16.0	− 0.5		− 0.7	− 0.1		− 0.5
		16.5	− 0.4		− 0.5	0.0		− 0.1
		17.0	− 0.3		− 0.3	0.1		0.1
	Mean difference		− 0.7		− 0.9	− 0.1		− 0.8

*FMI FFMI* and mean difference values are obtained by subtracting the own values from the reference values of the mentioned studies. Positive values indicate a higher value of the reference study compared to our study; negative values indicate a lower value

Difference in reference values for the referred age groups and mean difference between the reference studies and EVA4YOU study are provided for the 50th, 75th, and 90th percentiles. For Gätjens et al. only the 50th and 90th percentiles are provided because of missing reference values for the 75th percentile from the reference study

All values are presented in the unit kilograms per square meter

### Comparison between body composition classification based on FMI and BMI

**Fig. 2:**
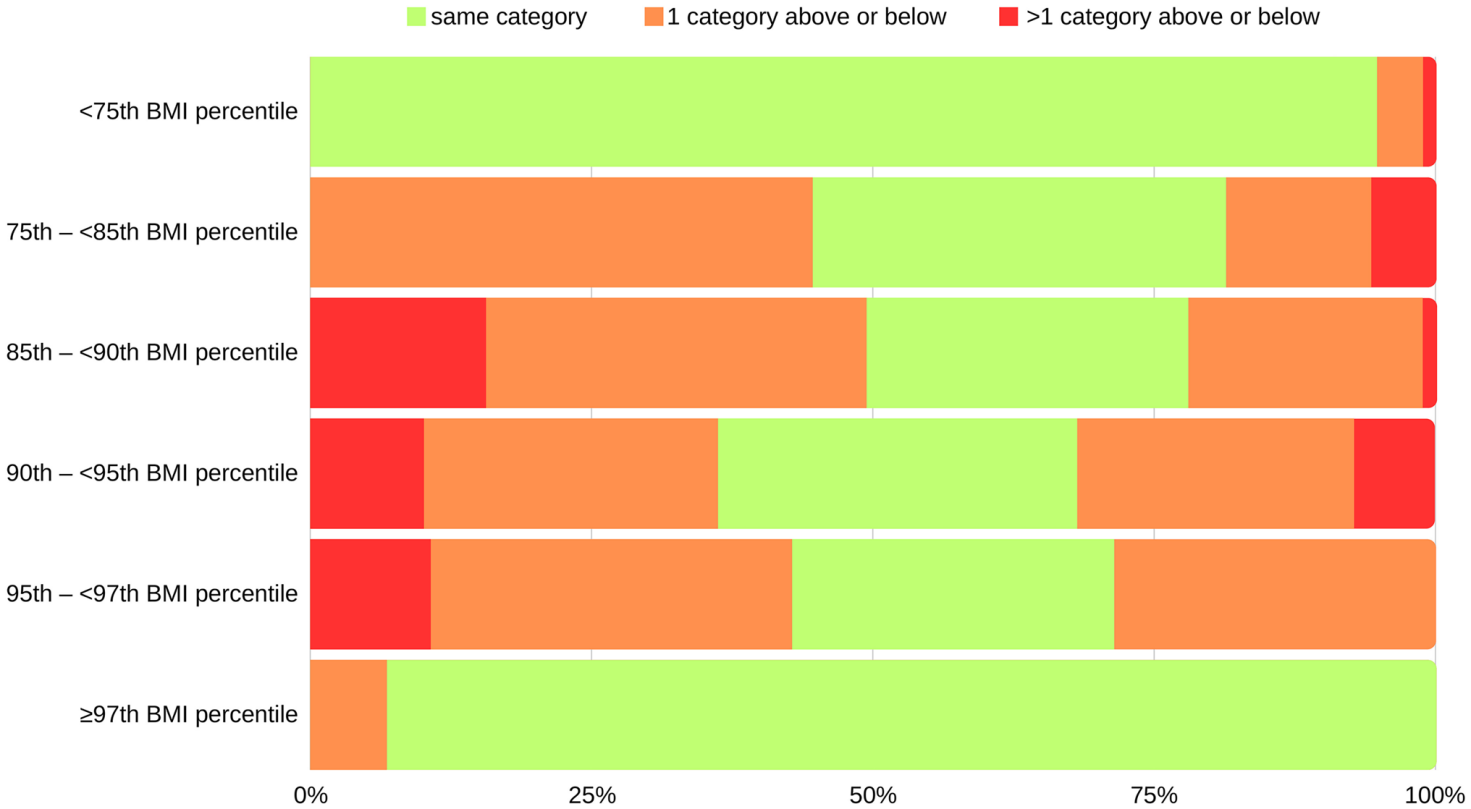
Change in categorization of body composition when FMI percentiles are used instead of BMI percentiles. The columns denote the population in different BMI categories (< 75, 75– < 85, 85– < 95, 95– < 97, ≥ 97th percentile). The color coding shows the proportion of the population that stays in the same category based on FMI (in green) or that changes by one (in orange) or two (in red) categories

Table 4 shows the percentage of re-categorization when FMI percentile categories of body composition are applied instead of those based on BMI. 94.7% of study participants with clearly normal BMI values below the 75th percentile had an FMI below the 75th percentile. Also, in the highly pathologic range of BMI over or at the 97th percentile, a majority of adolescents (93.2%) were in the same FMI group. The overlap of the categorization based on BMI and FMI was less pronounced in the four categories between the ≥ 75th and < 97th percentile with 36.7%, 28.6%, 31.9%, and 28.6% correctly classified in the 75th– < 85th, 85th– < 90th, 90th– < 95th, and 95th– < 97th percentile groups. In the whole study population, based on FMI measurements, 15.5% (221 of 1422) switch one category above or beyond those assigned by BMI and 3.4% (48 of 1422) change to more than one category higher or lower. Figure 2 visualizes the proportion of change in percentile category when FMI is used instead of BMI. In the subgroup of those between the 75th and 97th BMI percentiles, 24.9% (78 of 313) were categorized as a lower category than the 75th percentile and 4.5% (14 of 313) as a higher category than the 97th percentile based on FMI.

**Table 4:** Comparison of categorization in body composition based on BMI with FMI percentiles

		BMI												Total	
		< 75th percentile		75th– < 85th percentile		85th– < 90th percentile		90th– < 95th percentile		95th– < 97th percentile		≥ 97th percentile			
		*N*	%	*N*	%	*N*	%	*N*	%	*N*	%	*N*	%	*N*	%
FMI	< 75th percentile	1009	**94.7%**	62	44.6%	12	15.6%	4	5.8%	0	0.0%	0	0.0%	1087	76.4%
	75th– < 85th percentile	44	4.1%	51	**36.7%**	26	33.8%	3	4.3%	1	3.6%	0	0.0%	125	8.8%
	85th– < 90th percentile	8	0.8%	18	12.9%	22	**28.6%**	18	26.1%	2	7.1%	0	0.0%	68	4.8%
	90th– < 95th percentile	4	0.4%	8	5.8%	16	20.8%	22	**31.9%**	9	32.1%	0	0.0%	59	4.1%
	95th– < 97th percentile	0	0.0%	0	0.0%	0	0.0%	17	24.6%	8	**28.6%**	3	6.8%	28	2.0%
	≥ 97th percentile	0	0.0%	0	0.0%	1	1.3%	5	7.2%	8	28.6%	41	**93.2%**	55	3.9%
Total		1065	100.0%	139	100.0%	77	100.0%	69	100.0%	28	100.0%	44	100.0%	1422	100.0%

Values in bold indicate the percentage of agreement between the same BMI and FMI percentile categorization

When applying cruder BMI and FMI cut-offs for normal (< 85th percentile), overweight (85–95th percentile), and obesity (> 95th percentile), 3.2%, 46.6%, and 16.7% were reclassified by FMI compared to BMI, respectively.

When using commonly accepted cut-offs (i.e., BMI ≥ 85th [30] and FMI ≥ 75th [18]), 15.3% would be classified as obese based on BMI and 23.6% based on FMI.

### Comparison of FFMI z-score and BMI

To test the hypothesis that the cause of the discordant categorization by BMI and FMI is caused by differences in muscle mass, we compared the mean FFMI *z*-score in adolescents that were classified in the same category by both measures of body composition to those classified in a lower or higher category by FMI (Table 5). In those that were classified in a higher obesity category by the FMI than the BMI mean FFMI, *z*-scores were significantly lower (except for < 75th BMI percentile) and those who were categorized in a lower obesity category by FMI, the mean FFMI *z*-scores were significantly higher (except for ≥ 97th BMI percentile).

**Table 5:** Mean FFMI *z*-score of adolescents in the different groups classified by BMI percentiles in comparison with the groups that had matching group assignment based on BMI percentiles as well as FMI (“same classification”) and in the groups whose FMI was classified “below” or “above” the BMI percentile range

	Mean FFMI *z*-score^a^				
	Classified below	*p*-value^b^	Same classification	*p*-value^c^	Classified above
BMI < 75th percentile			− 0.34 (0.03)	0.170	− 0.44 (0.11)
BMI 75th– < 85th percentile	1.17 (0.05)	< 0.001	0.51 (0.05)	< 0.001	0.06 (0.07)
BMI 85th– < 90th percentile	1.30 (0.06)	< 0.001	0.81 (0.09)	< 0.001	0.15 (0.10)
BMI 90th– < 95th percentile	1.55 (0.08)	< 0.001	1.02 (0.09)	< 0.001	0.35 (0.09)
BMI 95th– < 97th percentile	1.85 (0.08)	< 0.001	1.32 (0.11)	0.010	0.93 (0.10)
BMI ≥ 97th percentile	2.00 (0.36)	0.322	1.86 (0.10)		

All categories were compared using ANCOVA, adjusted for sex and age, and bias corrected and accelerated

*BMI* body mass index, *FFMI* fat-free mass index

^a^Mean (SE), adjusted for sex and age

^b^*p*-value for the comparison of lower and same FMI classification

^c^*p*-value for the comparison of the same and higher FMI classification

**Fig. 3:**
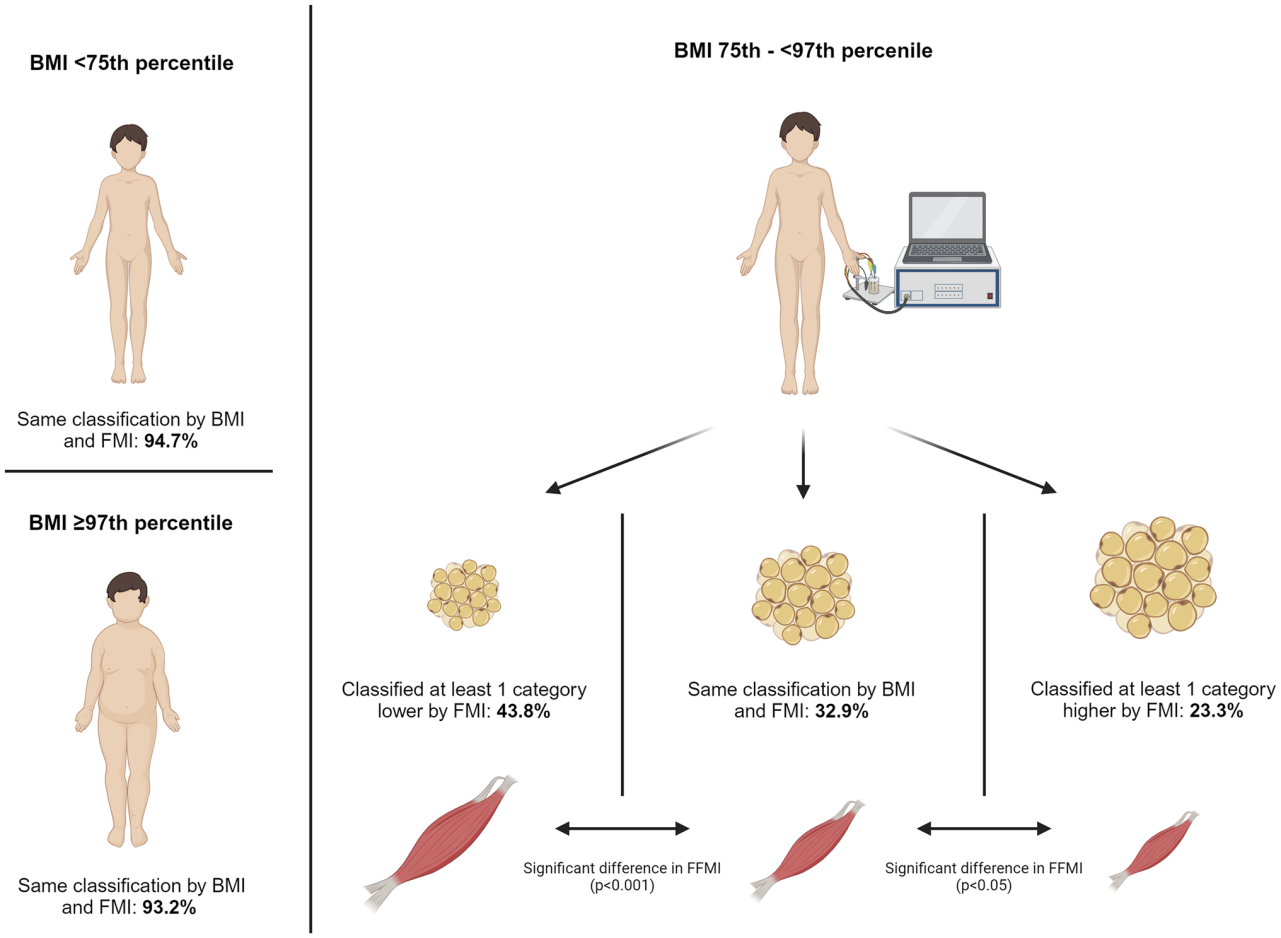
Graphic representation illustrating the differences in classification between body composition indices and BMI (created with https://www.biorender.com/)

A graphic representation of the main findings illustrating the differences in classification between body composition indices and BMI is shown in Fig. 3.

## Discussion

The current analysis allowed an in-depth insight into anthropometric measurements of a representative Tyrolean population of generally healthy adolescents. Although BMI is a simple and common tool for assessing overweight and obesity in clinical settings and epidemiological studies, the exclusive use of BMI to classify obesity as a risk factor for NCD in children and adolescents may lead to misclassification because the distinction between fat mass and fat-free mass is not considered. So far, these parameters were mainly measured by dual-energy X-ray absorptiometry (DXA) [18, 32, 33] posing a certain X-ray exposition and thereby limiting its clinical applicability, especially in children. In contrast, BIA is a non-invasive and safe method of body composition analysis that uses a small electrical current to estimate body fat and muscle mass and is a simple and accurate alternative to DXA [34–36]. A broad application of fat mass assessment could help to diagnose obesity more accurately and fat mass could potentially show a closer association with metabolic complications. However, it is unclear if reference data for FMI and FFMI in children and adolescents from Northern Germany, South-east England, or America are valid for Austria as well. When comparing the percentiles generated from these cohorts to our large representative sample of Tyrolean adolescents aged 14–19 years, we found good alliance with the German and English reference values. Relevant differences to the American percentiles can likely be attributed to varying lifestyle factors such as dietary habits, physical activity, and the geographical location on a different continent with distinct cultural and genetic influences as well as on the different measurement methods with DXA.

In addition, we explored differences in classification in normal to highly pathologic values based on BMI or FMI percentiles. When using suggested cut-offs for obesity (85th percentile of BMI [30] and the 75th percentile FMI [18]), 8.3% more adolescents of our population (15.3% vs. 23.6%) would have been classified as obese when using FMI instead of BMI. Yet, categorization of BMI [30, 31] is inconsistent and categorization using FMI was suggested without a biological or pathophysiological basis [37]. Therefore, we used various cut-offs (75th, 85th, 90th, 95th, and 97th population-specific percentile) for both BMI and FMI. We could show that there is good (> 90%) accordance in classification in the clearly normal (< 75th percentile) and the clearly pathologic (≥ 97th percentile) range. In the four remaining categories, less than one-third remained in the same category when categorized by BMI and FMI and about 7% even differed in more than one category.

Our findings show that the application of FMI—as a measurement of body fat—leads to a substantially different categorization of adiposity in 18.9% (269 out of 1422) (Table 4) of our population compared to BMI—that only considers body size and weight. An obvious explanation is that BMI does not consider the muscle mass. For example, a muscular athlete will yield a high body weight based on an overproportional muscle mass and considerable low body fat, whereby an adolescent with very low muscle mass will be categorized with a normal BMI because of a considerable amount of body fat. This common notion is supported by our data showing significantly higher or lower FFMI values when adolescents were classified over or under the category attributed to by BMI (Table 5).

Our results confirm, generalize, and expand the findings from a small pilot study with similar design on 380 kindergarten and elementary school children with high levels of physical activity comparing BIA FMI measurements with BMI. Farbo and Rhea found that less than 10% of non-overweight (FMI/BMI < 85th percentile) and obese (FMI/BMI > 95th percentile) were reclassified by FMI compared to BMI, yet this was the case in 38% of overweight children [38].

Still, due to the different reference values for FMI and FFMI in the different studies with different methods (four-compartment model, DXA, BIA) further validating studies are warranted to explore impedance (BIA) as an alternative to DXA in pediatric populations. However, recent studies advocate the use of BIA, both clinically and scientifically, to determine fat mass in children and adolescents rather than DXA [34–36], especially considering the radiation exposure of the latter method. Our data is limited in a way that we were not able to span the whole age range of adolescence (10–19 years, as defined by the World Health Organization [39]), but report only details on those aged 14 to 19 years.

The strengths of our study population include the large, representative, and well-characterized study cohort of generally healthy adolescents of a central European country with adolescents from general secondary schools, vocational schools, and already working adolescents with different sports and eating habits, both of rural and of urban origin, as well as a representative average of different ethnicities of a central European country. Furthermore, we show age- and sex-specific data.

In summary, reference values for FMI and FFMI are very similar in European cohorts and might therefore, pending further validation in Southern and Eastern European populations, be generalizable for the whole continent. The use of FMI reclassifies a relevant number of adolescents when compared to the BMI and might therefore be more suitable to define obesity. Yet, before FMI can broadly be implemented in clinical routine testing, cut-off values differentiating normal from pathologic FMI values on a biological basis are needed.

## Data Availability

No datasets were generated or analyzed during the current study.

## Abbreviations

BIA: Bioelectrical impedance analysis
BMI: Body mass index
EVA4YOU: Early Vascular Ageing in the YOUth
FFM: Fat-free mass
FFMI: Fat-free mass index
FM: Fat mass
FMI: Fat mass index
NCD: Non-communicable diseases

## Declarations

### Disclaimer

The funder had no role in the design and conduct of the study; collection, management, analysis, and interpretation of the data; preparation, review, or approval of the manuscript; and decision to submit the manuscript for publication.
